# BRD4: a general regulator of transcription elongation

**DOI:** 10.1080/21541264.2022.2108302

**Published:** 2022-09-01

**Authors:** Elisabeth Altendorfer, Yelizaveta Mochalova, Andreas Mayer

**Affiliations:** a Otto-Warburg-Laboratory, Max Planck Institute for Molecular Genetics, Berlin, Germany; b Department of Biology, Chemistry and Pharmacy, Freie Universität Berlin, Berlin, Germany

**Keywords:** RNA polymerase II, transcription elongation, promoter-proximal pausing, BET proteins, BRD4, PROTAC

## Abstract

Transcription elongation by RNA polymerase II (Pol II) has emerged as a regulatory hub in gene expression. A key control point occurs during early transcription elongation when Pol II pauses in the promoter-proximal region at the majority of genes in mammalian cells and at a large set of genes in *Drosophila*. An increasing number of *trans*-acting factors have been linked to promoter-proximal pausing. Some factors help to establish the pause, whereas others are required for the release of Pol II into productive elongation. A dysfunction of this elongation control point leads to aberrant gene expression and can contribute to disease development. The BET bromodomain protein BRD4 has been implicated in elongation control. However, only recently direct BRD4-specific functions in Pol II transcription elongation have been uncovered. This mainly became possible with technological advances that allow selective and rapid ablation of BRD4 in cells along with the availability of approaches that capture the immediate consequences on nascent transcription. This review sheds light on the experimental breakthroughs that led to the emerging view of BRD4 as a general regulator of transcription elongation.

## Introduction: regulation of Pol II transcription elongation

RNA polymerase II (Pol II) is responsible for transcription of all protein-coding and a large set of non-coding genes in the nucleus of eukaryotic cells [1–3]. Pol II transcription is generally subdivided into three main phases: initiation, elongation, and termination [4,5]. During the initiation phase, Pol II is recruited to the gene promoter and begins with RNA synthesis at the transcription start site (TSS) [6–8]. Pol II then transitions into the elongation phase where a nascent RNA is produced in a processive way by a stable transcription elongation complex [9–11]. At the 3’-end of genes, at the polyadenylation (pA) site, the transcript is cleaved and then polyadenylated [12,13]. RNA cleavage is a requirement for efficient transcription termination that occurs in a region downstream of the pA site [14–16], called the termination zone, where Pol II dissociates from the DNA template and transcription ends [17,18]. A new cycle of transcription can be re-initiated.

All three stages of the transcription cycle are points of regulation. The established view that transcription initiation represents the major regulatory step in transcription was challenged by the discovery of widespread transcriptional pausing in the promoter-proximal region of a large set of genes in *Drosophila* and at the vast majority if not all genes in mammalian cells [19–26]. Promoter-proximal pausing functions as a major checkpoint in early elongation before Pol II commits to productive transcription elongation [5,27–30]. At this decision point Pol II either continues elongation or terminates prematurely [31–33]. The determinants that underlie this decision have remained unclear.

Promoter-proximal pausing is regulated by a growing list of *trans*-acting factors and *cis*-DNA motifs. A misregulation of promoter-proximal pausing leads to altered gene expression and can contribute to disease development [34,35]. Transcriptional pausing is not restricted to the promoter-proximal region of genes, but occurs throughout the transcription unit providing additional potential points for regulation during the elongation phase [36–38]. Transcription elongation is tightly coordinated with co-transcriptional RNA processing including 5’-RNA capping, splicing and 3’-end RNA processing [34,39–45]. Changes in the elongation velocity such as by transcriptional pausing can create time windows for the kinetic coupling of transcription with co-transcriptional mRNA maturation [17,46–48]. However, key aspects of this tight coordination of processes in cells have remained obscure.

BRD4 has been implicated in the regulation of Pol II transcription elongation [49,50]. BRD4 belongs together with BRD2, BRD3 and BRDT to the mammalian BET bromodomain protein family [49,51,52]. BRD2/3/4 are ubiquitously expressed in all mammalian cell and tissue types investigated so far [53,54], whereas the expression of BRDT is restricted to testis [55]. Homologues of mammalian BET proteins also exist in flies and yeast [52,56]. BRD4 has been implicated in a broad range of human diseases including different types of cancer [57–62]. The overall domain organization of BET proteins is very similar. BET proteins possess two bromodomains (BDs) and an extra-terminal (ET) domain representing a protein–protein interaction domain [63–67]. BET bromodomains bind to acetylated chromatin and are likely required for the recruitment of BET proteins to their target genes [53,68–72], although a bromodomain-independent recruitment mechanism was also proposed [73]. BET bromodomain inhibitors and recently also BET protein degraders are evaluated as putative treatments of different types of cancers [74–78]. Immediate BRD4-specific roles in transcription and especially in elongation have only recently emerged.

This review sheds light on the key experiments performed and model systems used that have started to reveal direct BRD4-specific functions in transcription elongation control in mammalian cells.

## Conventional model of BRD4 function in Pol II transcription control

**Figure 1.**
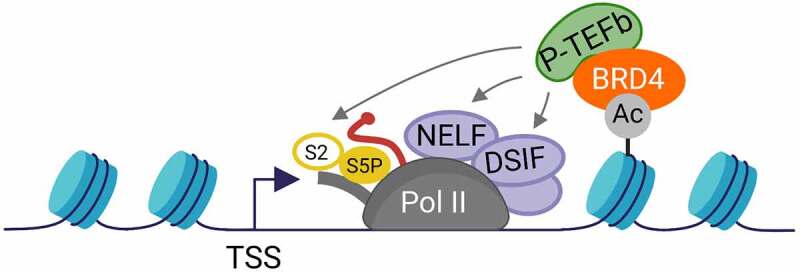
**Classic model of the role of BRD4 in Pol II transcription regulation**. BRD4 recruits P-TEFb to the promoter-proximal gene region through its binding to acetylated chromatin. Chromatin-bound P-TEFb phosphorylates different components of the Pol II transcription machinery indicated by gray arrows, leading to pause release. The DNA and nascent RNA are indicated as dark blue or red lines, respectively. The CTD is indicated as a gray tail of Pol II. Phosphorylation is depicted as a yellow bubble. TSS: transcription start site; Ac: Acetylation; (adapted from [79]).

According to the classic model of the role of BRD4 in Pol II transcription, BRD4 binds to acetylated chromatin and recruits the positive transcription elongation factor b (P-TEFb) to the promoter-proximal region of genes (Figure 1) [79]. P-TEFb consists of the cyclin-dependent kinase 9 (CDK9) and one of the corresponding Cyclins T1, T2 or K [80]. Once recruited to the chromatin, P-TEFb phosphorylates various components of the Pol II transcription machinery including the C-terminal repeat domain (CTD) of the largest subunit of Pol II (Rpb1), the SPT5 subunit of the DRB sensitivity-inducing factor (DSIF) complex and the NELF-E subunit of the negative elongation factor (NELF) complex [9,81–83]. The mammalian CTD consists of 52 heptapeptide repeats of the consensus amino acid sequence Tyr1-Ser2-Pro3-Thr4-Ser5-Pro6-Ser7 [84–86]. P-TEFb (CDK9) can phosphorylate the CTD at Ser2 residues in cells and the CTD Ser2-phosphorylated form is considered as the elongating form of Pol II [84–87]. These phosphorylation events together lead to the dissociation of NELF from the chromatin, a conversion of DSIF from a negative to a positive elongation factor that accompanies transcribing Pol II, the breakage of contacts with the Mediator complex and ultimately to the release of paused Pol II into productive elongation [27,28,30,88–91].

**Figure 2.**
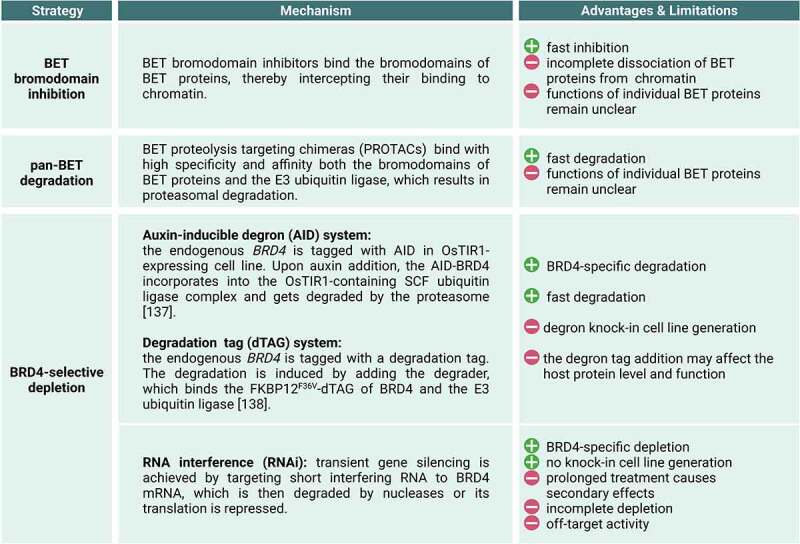
**Experimental strategies applied to perturb BRD4 in mammalian cells.** BRD4 protein-specific depletion methods include the auxin inducible degron (AID) (137) and the degradation tag (dTAG) (138) systems.

The conventional view that the main function of BRD4 in transcription control lies in the recruitment of P-TEFb to promoter-proximally paused Pol II [79] was mainly based on the following experimental evidence: (1) Epitope-tagged versions of BRD4 that were ectopically expressed from vectors in HeLa cells co-purified with the P-TEFb subunits CDK9 and cyclinT1 indicating an interaction [92,93]. (2) Transfection of HeLa cells with BRD4 increased luciferase reporter gene expression in a dose-dependent manner that was under the control of the HIV-1 long terminal repeat (LTR) promoter or of the c-MYC or c-JUN promoters. This suggested that BRD4 positively regulates transcription from these promoters [92]. (3) *In vitro* transcription assays with nuclear extracts obtained from HeLa cells that were simultaneously depleted of BRD4 and P-TEFb showed that transcription could only be fully restored if both factors were added back. This provided *in vitro* evidence for a role of BRD4 in transcription that is dependent on P-TEFb [93]. (4) Chromatin immunoprecipitation (ChIP) of P-TEFb coupled with real-time PCR upon >48 h RNAi-mediated BRD4 knockdown (Figure 2) at the HIV1-LTR reporter gene led to a reduction in the P-TEFb occupancy at the reporter locus [92]. The authors concluded that BRD4 is required for the recruitment of P-TEFb to the reporter gene.

Although both seminal studies established first direct implications of BRD4 in Pol II transcription, they retrospectively also suffered from limitations [92,93]. First, experiments were performed under ectopic overexpression of BRD4 at non-physiological BRD4 protein levels. Second, analyses were conducted at reporter genes or *in vitro*, and not at endogenous genes located in the native chromatin environment. Third, given the long depletion time of BRD4 in RNAi experiments of several days it remained unclear whether the reduction of P-TEFb occupancy at the chromatin was a direct consequence of BRD4 depletion or rather an indirect effect (Figure 2). Finally, findings were exclusively obtained for single reporter genes. Generalizations of observations to endogenous genes or larger gene sets are therefore difficult.

Due to these limitations, the classic model was rather speculative, especially the implication of BRD4 in Pol II pause release and elongation. However, it is still the predominant view in the field. This model was recently challenged by new studies that investigated direct roles of BET proteins and of BRD4 in transcription regulation as described in the following sections.

## BET inhibition and induced degradation reveal a general role of BET proteins in transcription elongation

The discovery of BET proteins as potent targets in cancer therapy led to the establishment of pan-BET bromodomain inhibitors and pan-BET protein degraders (Figure 2). In a collaborative effort, JQ1, a small cell permeable molecule, has been developed. JQ1 is an acetyl-lysine mimetic and binds specifically to the hydrophobic acetyl-lysine pocket of BET bromodomains, thereby displacing BET proteins from the chromatin [94]. Strikingly, JQ1 treatment of patient derived squamous carcinoma cells expressing the BRD4-NUT oncoprotein led to cell differentiation, arrest of proliferation and induction of cell death highlighting its potency as potential anti-tumor drug [94]. Besides JQ1, additional inhibitors of BET bromodomains, I-BET and I-BET151, were developed [95,96]. In addition to the potency of pan-BET bromodomain inhibitors for tumor and anti-inflammation therapy [97], their potent action also opened the doors for detailed studies of the role of BET proteins in transcription.

Exposure of multiple myeloma cells (MM1.S) for 6 hours to 500 nM JQ1 revealed a genome-wide reduction of the occupancy of BRD4, the Mediator and P-TEFb predominately at enhancers; however, the most pronounced loss has been observed at super-enhancer regions [98]. Similar observations were obtained for human and murine embryonic stem cells upon pan-BET inhibition [99]. Super-enhancers are clusters of enhancers, which are occupied by a high density of transcription factors and components of the Pol II transcription machinery including BRD4 and Mediator, mainly controlling cell identity genes [100,101]. By Pol II ChIP-seq analyses upon JQ1 treatment, the study also found a decrease in the density of Pol II across the elongating region of the gene-body at 50% of active genes suggesting an elongation defect [98]. Consistently, other studies performed in murine heart tissue, human MOLT4 cells and G1E-ER4 cells revealed an increase in the pausing index upon JQ1 treatment at selected genes and genome-wide suggesting an elongation defect [76,102,103]. The pausing index is a metric that has been widely used to estimate the impact on elongation by comparing the Pol II density in the promoter-proximal and the gene-body region [20,104]. An increase in the pausing index suggests an increase in promoter-proximal pausing or a decrease in productive elongation, or both. Inhibition of BET bromodomain function by the inhibitor I-BET151 impaired the recruitment of BRD4, P-TEFb, and PAF1 to the TSS of selected genes in human HL60 leukemia cells, and provided evidence for an interaction of BRD4 with the super elongation complex [96]. I-BET151 treatment displaced BET proteins mainly downstream of the TSS which correlated with increased promoter-proximal pausing of Pol II in human K562 and MV4-11 cells [105].

Although pan-BET bromodomain inhibitors have been a powerful tool for the analysis of the molecular mechanisms of transcription regulation, they also have limitations (Figure 2) [49]. One main limitation is that BET bromodomain inhibition results in incomplete phenotypes masking the cellular roles of BET proteins. This is mainly because of the following reasons. First, BET inhibitors only target and disrupt the function of the BET bromodomain leaving the other domains including the ET domain intact. Second, BET inhibition only leads to a partial dissociation of BET proteins from the chromatin [106].

To overcome limitations of BET inhibition, BET protein-specific degraders have been developed. BET degraders like the first-generation dBET1 and the improved second-generation dBET6, outperform BET inhibitors (JQ1) in terms of potency, efficiency and kinetics [76–78,107]. Degraders are proteolysis targeting chimeras (PROTACs) that couple the inhibiting properties of JQ1 to the ubiquitin E3 ligase complex, leading to ubiquitination and proteasomal degradation of BET proteins (Figure 2) [76,107–109]. Pan-BET degraders lead to an elimination of the entire BET proteins allowing the analysis of cellular phenotypes in the almost complete absence of BET proteins.

**Figure 3.**
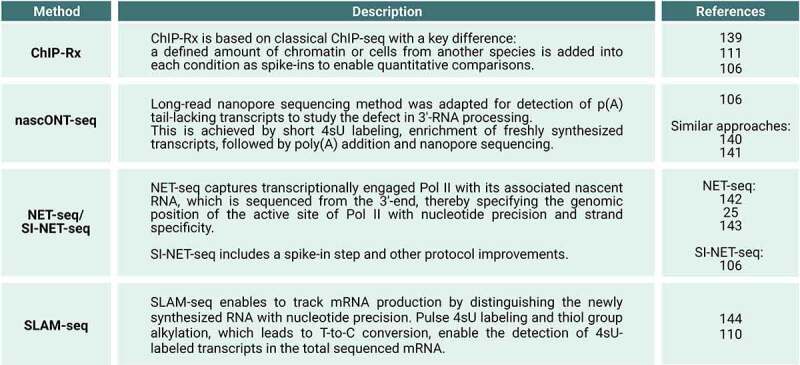
**Methods used to study the immediate impact of BRD4 perturbation on transcription.** ChIP-Rx: chromatin immunoprecipitation with reference exogenous genome (106, 111, 139); nascONT-seq: nascent Oxford nanopore sequencing (106) and similar approaches (140, 141); NET-seq: native elongating transcript sequencing (25, 142, 143); SI-NET-seq: spike-in controlled NET-seq (106); p(a): polyadenylation; SLAM-seq: thiol(SH)-linked alkylation for the metabolic sequencing of RNA (110, 144); T: thymine; C: cytosine; 4sU: 4-thiouridine.

While JQ1 treatment displaces BRD4 preferentially from super-enhancer regions and therefore perturbs mainly super-enhancer driven gene expression [98], exposure of human MOLT4 cells with 100 nM dBET6 uncovered a widespread reduction of the transcriptional output of genes within 2 hours [76]. Interestingly, dBET6 treatment induced a global collapse of transcription elongation as detected by a decrease in the level of transcriptionally engaged Pol II and of the Pol II CTD Ser2-phosphorylated form in the gene-body using native elongating transcript sequencing (NET-seq) and ChIP-Rx, respectively (Figure 3) [76]. Notably, this elongation defect was independent of CDK9 (P-TEFb) recruitment [76]. Similar results were obtained by dBET6 treatment of glioblastoma cells (U87, U251), showing defects in Pol II transcription elongation in combination with a reduction of active histone marks (H3K27Ac, H3K4me3) [77].

Although pan-BET inhibition and degradation point toward a role of BET proteins in elongation control, direct BRD4-specific functions have remained unclear.

## Direct roles of BRD4 in transcription elongation

Three recent studies illuminate direct BRD4-specific functions in the regulation of Pol II transcription elongation in human cells [106,110,111]. All studies combined rapid and BRD4-selective degradation in cells with approaches that capture the immediate consequences on Pol II transcription before the phenotype is overlaid with indirect effects. Muhar et al. and Zheng et al. used the auxin-inducible degradation (AID) system to rapidly deplete BRD4 in human K562 and DLD-1 cells, respectively (Figure 2). Arnold et al. inserted a degradation tag (dTAG) into the endogenous BRD4 gene in K562 cells and induced degradation with a PROTAC degrader (Figure 2) [106]. The degron-tagged versions of BRD4 were expressed from the endogenous locus under the control of the natural promoter. The splicing of the BRD4 transcript results in protein-coding long (BRD4-L) and short (BRD4-S) isoforms [112,113]. Both isoforms contain the same two bromodomains and, therefore, can bind to acetylated lysines of histones and transcription factors. In Muhar et al. and Arnold et al. levels of the long and the short BRD4 protein isoforms were almost completely eliminated within less than two hours [106,110]. In Zheng et al., only the long BRD4 protein isoform was rapidly depleted [111]. Rapid BRD4 protein degradation has a significantly higher kinetic resolution compared to previous RNAi-mediated BRD4 mRNA depletion that required prolonged treatment times of several days (Figure 2).

All three studies provide consistent evidence that acute BRD4-specific ablation disrupts pause release of Pol II into productive elongation. Muhar et al. [110] performed ChIP-Rx experiments of two different CTD phosphorylated forms of Pol II to assess the immediate consequences upon rapid BRD4-selective degradation on transcription (Figure 3). This study found that the Pol II CTD Ser5-phosphorylated (Ser5-P) form accumulated in the promoter-proximal region, whereas the Pol II CTD Ser2-P form decreased over the gene-body suggesting a defect in pause release. Consistently, SLAM-seq upon acute BRD4 loss revealed a global reduction of newly synthesized RNAs (Figure 3). Zheng et al. [111] performed ChIP-Rx of total Pol II upon auxin-inducible degradation of the long BRD4 protein isoform revealing an increase of the Pol II density in the promoter-proximal region coupled with a reduction along the gene-body. Arnold et al. [106] developed and applied an improved spike-in NET-seq approach, called SI-NET-seq, which allowed quantitative comparisons of nascent transcription between conditions and cellular states (Figure 3). SI-NET-seq showed that acute BRD4 loss led to an immediate accumulation of transcriptionally engaged Pol II in the promoter-proximal region and to a decrease of transcribing Pol II over the gene-body region indicating a defect in elongation activation. These findings together suggest that the general defect in pause release of Pol II was a direct consequence of acute BRD4 loss.

Notably, P-TEFb (CDK9) occupancy was not affected by BRD4 ablation [106,110,111] confirming an earlier observation that BET proteins are likely not involved in the recruitment of P-TEFb [76].

Arnold et al. [106] provided additional insights into the molecular mechanisms of early elongation control by BRD4. Acute BRD4 loss also led to an immediate reduction of the PAF1 complex (PAF) at genes as revealed by ChIP-Rx (Figure 3). This finding was consistent with an earlier observation that pan-BET inhibition can reduce PAF levels at the 5’-end of selected genes [96]. Notably, the reduction was more pronounced than the overall decrease of the Pol II occupancy at the promoter-proximal region of genes upon acute BRD4 ablation indicating a recruitment defect of PAF during early transcription elongation [106]. PAF represents an integral component of the Pol II elongation complex [9,114,115]. Furthermore, Arnold et al. [106] provided evidence with quantitative proteomics that BRD4 interacts with other elongation factors including SPT5 (DSIF) and SPT6. Together, these results suggest that BRD4 is required for the assembly of the Pol II elongation complex at the 5’-end of genes.

Using the SI-NET-seq approach the study further found that the rapid loss of BRD4 caused a global transcriptional readthrough, indicating the failure of Pol II to timely terminate transcription in a region downstream of the pA site [106]. Interestingly, the transcriptional readthrough led to extended transcripts as revealed by nascONT-seq (Figure 3). Extended transcripts at the readthrough genes are indicative for 3’-end RNA cleavage defects. Finally, the study found that the occupancy of the CstF and CPSF complexes, representing the key components of the general 3’-end RNA processing machinery [13], was strongly decreased at the promoter-proximal region of genes as shown by ChIP-Rx. This decrease of occupancy occurred at the same promoter-proximal location where BRD4 predominantly binds along genes [98,106,116,117]. The reduction of CstF and CPSF levels was more pronounced than the overall decrease of Pol II density upon BRD4 degradation in this region. Consistently, an interaction of BRD4 with several subunits of the 3’-end RNA processing machinery could be identified and acute BRD4 loss provoked immediate displacement of 3’-end RNA processing factors from the chromatin as revealed by quantitative proteomics analyses. Together, these findings suggest that BRD4 is involved in the recruitment of 3’-end RNA processing factors during the 5’-elongation control point [106].

The impaired recruitment of 3’-end RNA processing factors during the 5’-elongation control point upon acute BRD4 ablation represents a likely cause for the detected 3’-end RNA cleavage and termination defects [106]. The observation that the extent of readthrough transcription upon BRD4 loss was highly similar to the massive transcriptional readthrough observed upon rapid depletion of CPSF73 [106,118], the endonuclease subunit of the CPSF complex which cleaves the nascent RNA [119], supports this view. However, it cannot be ruled out that the earlier elongation defects contribute to the later readthrough transcription.

**Figure 4.**
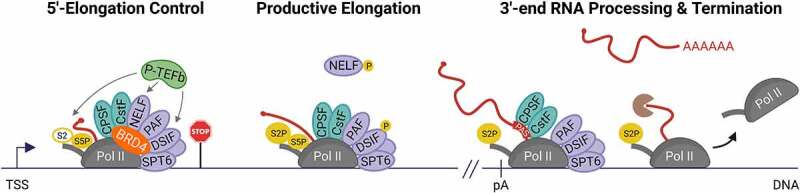
**Emerging direct functions of BRD4 in Pol II transcription elongation control**. BRD4 helps to assemble a functional Pol II elongation complex and recruits 3’-end RNA processing factors (CPSF, CstF) during a general 5’-elongation control point to allow productive elongation and proper RNA processing at the 3’-end of genes. The scheme also includes the SPT5 (DSIF) phosphorylation cycle [131–133]. Although elongation factors can contact the Pol II CTD and nascent RNA they are shown at a different location for clarity. Although NELF and PAF cannot bind to Pol II at the same time *in vitro* [9], the model depicts both factors to illustrate that they interact with BRD4 and are present at the promoter-proximal region of genes in cells [114,134–144]. Despite accumulating evidence that BRD4 binds to acetylated chromatin (Figure 1) it is not shown for clarity. The color code for nascent RNA, DNA and phosphorylations is as in Figure 1. The Pol II CTD and P-TEFb phosphorylation targets are indicated as in Figure 1. The torpedo termination factor XRN2 is shown as a brown pac-man. TSS: transcription start site; S2: Serine 2 residue of the CTD; S5P: phosphorylated serine 5 residue of CTD, S2P: phosphorylated serine 2 residue of the CTD; P: phosphorylation; pA: polyadenylation site; PAS: polyadenylation signal.

These findings and previous observations converge on the following proposed model of direct elongation control by BRD4 (Figure 4). BRD4 localizes to the promoter-proximal region of genes. At the target gene BRD4 helps to assemble a functional elongation complex at the 5’-end of genes. During this general 5’-elongation control point BRD4 also recruits 3’-end RNA processing factors. This allows the release of paused Pol II, productive elongation, proper 3’-end RNA processing, and transcription termination.

Since BRD4 interacts with both elongation factors (PAF, SPT5, SPT6 and others) and general 3’-end RNA processing factors (CPSF, CstF), and given the immediate impact on elongation, 3’-end RNA processing and transcription termination upon acute BRD4 loss it is likely that the functions of BRD4 in these processes are connected. Moreover, observations that the BRD4 interactors PAF and SPT5 are themselves implicated in 3’-end RNA processing and transcription termination [38,120–123] further illustrate the tight links between elongation, 3’-end RNA cleavage and termination in cells. Therefore, it will be difficult to uncouple the individual roles of BRD4 in these physically and kinetically linked processes.

## Summary and future directions

From these recent studies it becomes clear that BRD4 plays a more versatile role in transcription control than originally thought. BRD4 emerges as a general component of the Pol II transcription machinery that is required for elongation activation allowing global nascent RNA synthesis in mammalian cells (Figure 4). Notably, several recent studies independently provide evidence that BRD4 is likely not required for the recruitment of P-TEFb to genes in cells, challenging long-standing views. In addition to pause release, BRD4 serves as a molecular link between Pol II transcription and co-transcriptional RNA processing. More specifically, BRD4 helps to recruit 3’-end RNA processing factors during the 5’-elongation control point to allow proper 3’-end RNA processing and transcription termination (Figure 4).

Despite the strong recent progress in understanding the roles of BRD4 in transcription control, a complete picture has not yet emerged. Future work is required to clarify how BRD4 controls elongation activation and the transcriptional output of genes in cells. First of all, the functional interactions of BRD4 with other elongation factors, the Pol II machinery and chromatin regulators that are required for activation of productive elongation need to be identified. This includes the clarification of the relationship between BRD4 and P-TEFb, and with other elongation factors such as DSIF and PAF in Pol II pause release. Second, since different BRD4 protein isoforms are co-expressed in mammalian cells, the elucidation of BRD4 isoform-specific functions in transcription elongation represents an interesting future direction. Third, given that BRD4 co-localizes to enhancer regions, including super-enhancers [76,98,102,111,116,117,124–126], and interacts with the Mediator complex [92,127,128] more efforts are required to characterize its role in enhancer-target gene communication. Along these lines, BRD4 has been implicated in the formation of transcriptional condensates [129,130]. However, its direct role and molecular mechanisms in condensate formation in living cells have remained unclear. Finally, the new knowledge of BRD4-specific functions in elongation control can be applied to disease models to help decipher disease mechanisms.

The new enlarged multi-omics toolbox will continue to provide new and unexpected insights into the direct roles of BRD4 in fundamental cellular processes. A holistic understanding of BRD4-specific functions and its dysfunction in disease states holds the promise to open up therapeutic avenues for more selective treatments of human malignancies in future.
